# Erector spinae plane block spread patterns and its analgesic effects after computed tomography-guided hepatic tumour ablation: a randomized double-blind trial

**DOI:** 10.1080/07853890.2025.2480255

**Published:** 2025-03-19

**Authors:** Wei-Han Chou, Wen-Yun Niu, Po-Chin Liang, Shih-Han Lin, Jen-Ting Yang, Chih-Peng Lin, Ming-Shiang Wu, Chun-Yu Wu

**Affiliations:** a Department of Anesthesiology, National Taiwan University Hospital, Taipei, Taiwan; b Department of Medical Imaging, National Taiwan University Hospital Hsinchu Branch, Hsinchu, Taiwan; c Department of Anesthesiology, National Taiwan University Hospital Hsinchu Branch, Zhubei City, Hsinchu County, Taiwan; d Department of Anesthesiology & Pain Medicine, University of Washington, Seattle, WA, USA; e Department of Internal Medicine, National Taiwan University Hospital, Taipei, Taiwan

**Keywords:** Erector spinae plane block, living subjects, computed tomography, radiofrequency ablation

## Abstract

**Introduction:**

Spread patterns of the erector spinae plane block (ESPB) in a larger cohort of living subjects remain inadequately understood. This study investigated the spread of local anaesthetics or saline with contrast in patients undergoing computed tomography-guided radiofrequency ablation of hepatic tumours.

**Patients and methods:**

Thirty patients participated in a double-blinded randomized controlled trial, 14 April 2021 and 18 January 2023. These patients were randomized into two groups: the ESPB group, which received local anaesthetic with contrast, and the sham group, which received saline with contrast. The spread of the drug was assessed regarding vertebral levels and its correlation with the patient characteristics. Pain intensity and morphine consumption were also evaluated.

**Results:**

The ESPB consistently spread cranio-caudally to the dorsal erector spinae muscle in all patients, with a median (IQR) spread of 9 (8–11) vertebral levels, and to the intercostal space with a median (IQR) spread of 4 (3–6) vertebral levels. Paravertebral spread occurred in 90% of patients (27 out of 30) with a median (IQR) spread of 3 (2–5) vertebral levels, while epidural spread was observed in 36.7% of patients (11 out of 30) with a median (IQR) spread of 0 (0–2) vertebral levels. Cranio-caudal spread negatively correlated with back muscle thickness (*r*= −0.4; *p* = 0.035), and females exhibited significantly more intercostal spread levels than males (5.8 ± 1.0 vs. 4.3 ± 1.6 levels in females and males, respectively; *p* = 0.021). However, no significant difference was found in pain intensity and morphine consumption between the two study groups.

**Conclusion:**

This study provides insights into the drug spread patterns of ESPB in living subjects. However, a unilateral ESPB did not yield sufficient analgesic effects for radiofrequency ablation of hepatic tumours.

## Introduction

The erector spinae plane block (ESPB) is a relatively new regional anaesthesia technique that has gained popularity in clinical practice due to its technical simplicity, analgesic efficacy, and low incidence of complications [1]. However, there is controversy surrounding the drug spread pattern of ESPB, which is crucial for optimizing block performance and ensuring the safe and effective delivery of analgesia. Numerous cadaveric studies have focused on the issue of drug spread in ESPB. However, relying solely on these cadaveric studies is inadequate for establishing the actual drug spread pattern of this novel technique, as the drug spread in the fascial plane of living subjects can significantly differ from that observed in cadaveric models [2,3].

Computed tomography (CT)-guided percutaneous radiofrequency ablation (RFA) is a commonly used treatment option for hepatic tumours in patients who are not suitable for surgery. However, patients undergoing this procedure frequently experience post-procedural pain due to inflammatory responses and necrosis of the ablated hepatic tissues [4]. ESPB has been reported to reduce postoperative pain after laparoscopic cholecystectomy [5,6] and hepatic surgery [7]. Furthermore, ESPB has recently been reported in association with percutaneous ablation techniques like RFA or microwaves for hepatic tumour treatment [8,9]. Although literature supports the analgesic efficacy of ESPB for painful hepatobiliary procedures, its spread pattern has not been thoroughly investigated. Since patient-reported sensory loss following the block may not always accurately reflect the actual spread of ESPB [10], concurrently assessing both its analgesic effects and spread pattern could provide valuable clinical insights. Given that CT imaging is routinely performed during CT-guided RFA, this procedure offers a unique opportunity to investigate both the spread pattern of local anaesthetics following ESPB in living subjects and its analgesic efficacy during RFA, without exposing participants to additional radiation. Therefore, we hypothesized that ESPB would result in extensive drug spread in living subjects and provide effective analgesia for RFA in hepatic tumours. Consequently, we designed this double-blinded randomized controlled trial to evaluate its efficacy and distribution.

## Methods

### Study design, ethics and trial registration

This study was designed to investigate the spread of a 30 mL solution consisting of contrast with local anaesthetics or normal saline following a right ESPB block of T10 level, and the effects of post-procedural analgesia in patients who received computed tomography-guided radiofrequency ablation of hepatic tumours. This double-blinded, prospective, randomized, sham-controlled single-institution trial received approval from the Research Ethics Committee of National Taiwan University Hospital (Approval No. 202101007RINA) and written informed consent was obtained from all subjects participating in the trial. The trial was registered prior to patient enrolment at clinicaltrials.gov (NCT04837742; Principal investigator: Ming-Shiang Wu; Date of first submitted: 6 April 2021; First posted: 8 April 2021).

### Participants

The study recruited patients aged from 20 years to 85 years who were undergoing CT-guided hepatic tumour RFA with a tumour size larger than 2 cm between 14 April 2021 and 18 January 2023. Patients with the following conditions were excluded: a history of allergic reactions to local anaesthetics or contrast agents, abnormal kidney function, defined as an estimated glomerular filtration rate <30 mL min^−1^ 1.73 m^−2^, and coagulopathy. We conducted the study in accordance with the Declaration of Helsinki and its later amendments or comparable ethical standards, adhering to the applicable CONSORT guidelines.

### Randomization and blinding

Before the trial began, stratified randomization was performed by an independent statistical expert using a block size of 30 and 1:1 allocation. All patients provided written informed consent on the day before the RFA procedure to an investigator who was unaware of the randomization results. They were then equally and randomly assigned to either the ESPB group (patients who received the ESPB injection with local anaesthetics) or the sham group (patients who received the ESPB injection with an equivalent volume of normal saline). The masked drug was provided by an independent pharmacy, ensuring that the allocation was concealed from the investigators and clinicians. Therefore, the blinding was maintained for patients, clinical care providers, and the outcome investigators.

### Interventions and the rationales of the inclusion of sham group

This study was designed to include the sham group to differentiate between the physiological effects of ESPB, placebo-related effects and systemic analgesic effects of the fascial plane block [11] for improving the quality of blinding and reducing bias [12].

### Anaesthesia and ESPB

Each patient underwent standard intraoperative monitoring using a Philips IntelliVue MP70 monitor (Philips Medical Systems, Suresnes, France). After being placed in the left lateral decubitus position, the patients received 50 μg of intravenous fentanyl. The right-sided T10 spine level was identified by counting up from the L5/S1 junction, which was determined by an indentation of the reflective surface using the low-frequency curved probe. This level was chosen to potentially cover the insertion site, as well as the greater and lesser splanchnic nerves [13,14]. Before the procedure, pre-scanning was performed using both transverse and sagittal views to identify bony contours, including spinous processes, laminae, transverse processes, costotransverse junction, ribs, and erector spinae muscles at the right-sided T10 spine level. Upon recognizing the lateral edge of T10 transverse process, a 23-gauge needle (“NIPRO” KATERAN needle, 70 mm in length) was inserted in a caudal-to-cranial direction until it came into contact with the tip of the T10 transverse process. Next, the experimental drug was administered, which consisted of a mixture of 10 mL contrast media (Iohexol- Omnipaque^TM^, GE Healthcare, Chicago, IL, USA) with 20 mL 0.5% levobupivacaine for the ESPB group or a mixture of 10 mL contrast media (Iohexol- Omnipaque^TM^, GE Healthcare, Chicago, IL, USA) with 20 mL normal saline for the sham group, respectively. The distribution of the local anaesthetics was confirmed by observing the plane between the transverse process of the vertebra and the erector spinae muscle. After the completion of the ESPB, patients were sedated with a target-controlled propofol infusion (Schnider model) to maintain an effect site concentration of 2.5 to 3.5 μg/mL. During the RFA procedure, the attending anaesthesiologist, who was unaware of the group allocation, could administer opioids (fentanyl or remifentanil at the anaesthesiologist’s discretion) for the management of intraoperative analgesia.

Percutaneous RFA was performed under CT guidance in all patients by the same radiologist, using a single radiofrequency ablation electrode with a 200-W generator [15]. Subsequently, a low-dose CT scan with a minimalized scan range was obtained after adjusting each electrode. Before the RFA procedure, a whole spine CT scan was performed to evaluate the spread of ESPB in the following regions: erector spinae muscle plane, paravertebral space, intercostal space, and epidural space. All CT images were reviewed and analysed by the same independent pain specialist who had experience in interpreting spine CT images. Additionally, the thickness of the back muscle was measured to investigate potential correlations between the muscle thickness and the extent of ESPB spreads.

### Post-procedural pain assessment and management

Post-procedural pain intensity was assessed by investigators who were independent of the clinical care team. They utilized a 100-mm visual analogue scale (VAS) [13,14] to evaluate the highest pain intensity experienced by patients during either deep breaths or cough at 1 h and 24 h after the RFA procedure. During each assessment, both the investigator and the participant were blinded to the previous VAS questionnaire results to maintain objectivity. The results of the VAS were used for outcome analysis purposes only and were not utilized for clinical care decisions. To manage post-procedural pain, boluses of intravenous morphine (2 mg) were administered upon the patient’s request, aiming to maintain pain score < 4 (using 0–10 numeric rating pain score by the caring staff) in the post-anaesthetic care unit. For rescue analgesia, intravenous morphine (2–4 mg every 3 h) was provided upon patient request in the general ward. Additionally, oral acetaminophen was provided to each patient in the general ward every 6 h for 24 h following the RFA. Furthermore, the postoperative quality of recovery at 24 h was evaluated using the Quality of Recovery-15 (QoR-15) questionnaire [16].

### Outcomes

The primary outcomes of thIS study were to determine the drug spread pattern and the highest pain intensity, assessed using the 100-mm Visual Analogue Scale (VAS), at 24 h after the RFA procedure. Secondary outcome included the highest pain intensity at 1 hr, intraoperative fentanyl, postoperative analgesic usage and quality of recovery (QoR-15 score).

### Sample size calculation

Based on pilot data from patients undergoing RFA of hepatic tumours, the mean (SD) VAS score at 24 h after RFA was approximately 45 (30) mm. To detect a difference in mean VAS score of 35 mm (approximately a 80% reduction in pain intensity) with 80% power and a two-sided type I error of 0.05, a sample size of 26 patients (13 patients in each group) was calculated. To account for potential attrition, a total of 30 patients were enrolled in the study. This sample size was also considered adequate for evaluating the drug spread pattern, as it exceeded the number used in most previous studies investigating ESPB spread [3].

### Statistical analysis

The normality of the distribution was assessed using the Shapiro–Wilk test, and visual inspections were conducted using histograms. Continuous variables are presented as mean (standard deviation) or median (interquartile range), depending on the distribution. For dichotomous data, Fisher’s exact test or the chi-square test was used for analysis. Student’s *t*-test was applied to normally distributed continuous data, while the Mann–Whitney *U* test was used for nonparametric ordinal data. To investigate correlations between the numbers of vertebral spread levels and the back muscle thickness at the injection level, as well as patient characteristics like body mass index, Pearson’s correlation test was utilized. Statistical analyses were conducted using the PASS Sample Size Software (NCSS, LLC, Kaysville, Utah, USA) and MedCalc Statistical Software version 19.3.1 (MedCalc Software Ltd., Ostend, Belgium).

## Results

### Study population

**Figure 1.**
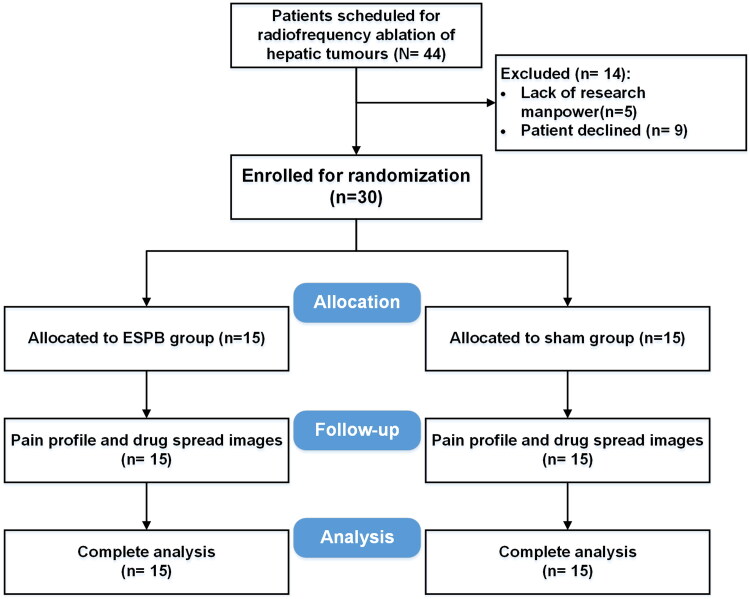
CONSORT diagram.

The original target sample size was set at 80 patients to identify potential differences in QoR-15 scores. However, enrolment was significantly impacted by the SARS-CoV-2 pandemic, and the final sample size was adjusted to focus on comparing the primary outcome, namely the difference in VAS scores, which requires a smaller sample size. Between August 2021 and May 2023, a total of 44 patients were initially assessed for inclusion in the study. After the screening process, 30 patients were ultimately included for the final analysis (see Figure 1). The baseline characteristics of the patients in the two study groups were comparable and showed no significant differences (Table 1). The time interval between the ESPB injection and the whole spine CT scan was 46.8 (11.8) min. However, it is worth noting that patients in the ESPB group had statistically non-significantly larger tumour size compared to the sham group (3.4 ± 1.6 cm in the ESPB group vs. 2.5 ± 1.0 cm in the sham group; *p* = 0.080).

**Table 1.** Patients characteristics in both of the study groups.

	ESPB (*n* = 15)	Sham (*n* = 15)	*p* value
Age (yr)	73 (69–77)	68 (63–78)	0.262
Sex (female; %)	4 (26.7%)	4 (26.7%)	1.000
Height (cm)	164 ± 11	162 ± 8	0.524
Weight (kg)	67.3 ± 9.6	67.7 ± 11.4	0.923
Body mass index	25.0 ± 2.6	25.8 ± 3.8	0.517
Comorbidity (*n*; %)			
Hypertension	7	8	1.000
Diabetes	5	6	1.000
Coronary arterial disease	1	2	1.000
Others	1	1	1.000
Tumour number (median; range)	1 (1–4)	1 (1–4)	0.827
Tumour size (cm; mean ± SD)	3.4 ± 1.6	2.5 ± 1.0	0.080

### Primary outcome analysis

#### Spread patterns of the erector spinae plane block

**Figure 2.**
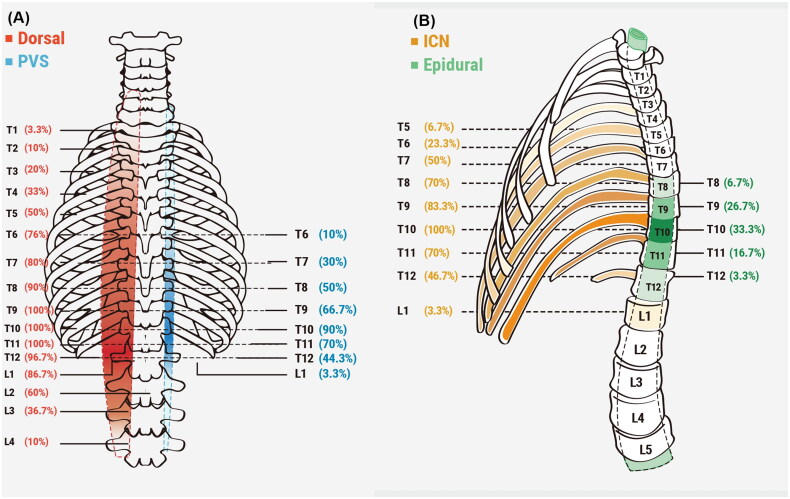
Schematic representation of the extent of dye spread; the numbers represent the proportion of patients with spread to the vertebral level. (A). Extent of intercostal spread and epidural spread. (B). Extent of cranio-caudal erector spinae muscle spread and paravertebral spread.

**Figure 3.**
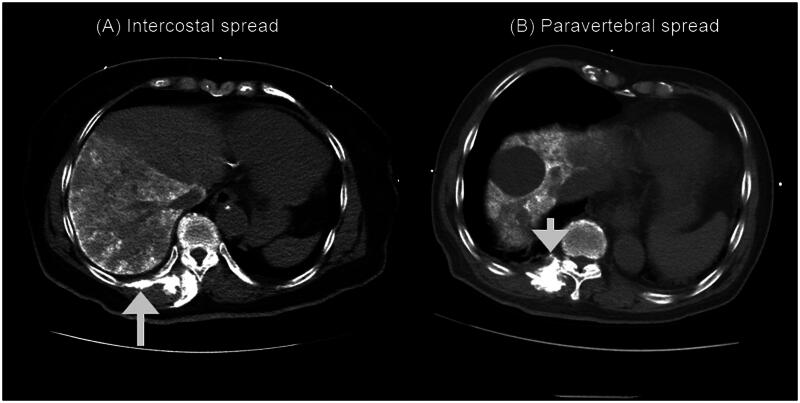
Computed tomography scan of the spine showing injectate spread. (A). Intercostal spread (upward arrow). (B). Paravertebral spread (downward arrow).

Figures 2 and 3 provide a summary of the profiles of drug spreads to the dorsal erector spinae muscle, intercostal space, paravertebral space, and epidural space. The cranio-caudal dorsal spread of the drug to the dorsal erector spinae muscle was observed in each patient, with a median (Q_1,_ Q_3_) spread of 9 (8–11) vertebral levels (Figure 2(A)). Similarly, the drug spread to the intercostal space was observed in each patient, with a median (Q_1,_ Q_3_) spread of 4 (3–6) vertebral levels (Figure 2(B)). The paravertebral spread was observed in 27 out of 30 (90%) patients, with a median (Q_1,_ Q_3_) spread of 3 (2–5) vertebral levels (Figure 2(A)). On the other hand, epidural spread was only observed in 11 out of 30 (36.7%) patients, with a median (Q_1,_ Q_3_) spread of 0 (0–2) vertebral levels (Figure 2(B)). Overall, there is a greater distribution of the contrast on the cranial side compared to the caudal side, starting from the needle insertion point (Figure 2). This distribution pattern aligns with the direction of the needle insertion. The CT images depicting these patterns of spread are illustrated in Figure 3.

The mean (SD) thickness of the back muscle at the T10 level was 34.3 (5.6) mm. The number of vertebral levels of cranio-caudal erector spinae muscle spread was negatively correlated with the thickness of the back muscle at the T10 level (*r* = −0.4; *p* = 0.035). However, there was no significant correlation between the number of vertebral levels of cranio-caudal erector spinae muscle spread and the number of epidural spread (*r* = −0.3; *p* = 0.076), the number of paravertebral spread (*r* = −0.4; *p* = 0.058), or the number of intercostal spread (*r* = −0.1; *p* = 0.528). Furthermore, the number of vertebral levels of intercostal spread was negatively correlated with the height of the patients (*r* = −0.5; *p* < 0.001), and the number of vertebral levels of epidural spread was negatively correlated with the weight of the patients (*r* = −0.4; *p* = 0.030). Additionally, it was observed that female patients had a significantly higher number of intercostal spread levels compared to male patients (5.8 ± 1.0 vs. 4.3 ± 1.6 levels in female and male patients, respectively; *p* = 0.021).

#### 24-hr pain intensity

There were no significant differences between the two study groups in terms of the highest VAS scores at 24 h after the RFA procedure.

#### Procedural data and secondary outcomes

Table 2 provides a summary of the procedural data and postoperative analgesic profiles of the two study groups. The procedural time was not significantly different between the ESPB group and the sham group [74.9 (34.4) min vs. 71.1 (29.5) min, respectively; *p* = 0.756]. However, patients in the sham group received a significantly lower median (Q1, Q3) fentanyl equivalent dose during the RFA procedure compared to the ESPB group [100 (100–215) μg vs. 78 (50–100) μg, respectively; *p* = 0.041]. Regarding postoperative analgesic profiles, there were no significant differences between the two study groups in terms of the highest VAS scores at 1 h and 24 h after the RFA procedure, the 24-h morphine dose, and the proportion of patients who requested no morphine (Table 2). Furthermore, the 24-h QoR-15 scores, which indicate postoperative quality of recovery, were comparable between the ESPB group and the sham group.

**Table 2.** Procedural data and postoperative analgesic profiles.

	ESPB (*n* = 15)	Sham (*n* = 15)	*p* value
Procedure time (min)	74.9 (34.4)	71.1 (29.5)	0.756
Intraoperative fentanyl equivalence (μg)	100 (100–215)	78 (50–100)	0.041
Highest VAS (0–100 mm)			
1 h	38 ± 39	37 ± 39	0.908
24 h	20 ± 26	17 ± 21	0.703
24-h morphine dose (mg)	1.7 ± 2.7	1.4 ± 1.6	0.747
Patient without morphine request (*n*; %)	10 (66.7%)	7 (46.7%)	0.462
Quality of recovery-15 score (0–150)	126 (83–139)	140 (99–144)	0.280

Data were mean ± SD or median (IQR). VAS = visual analogue scale.

## Discussion

In this study, we demonstrated the drug spread patterns in a relatively large series of living subjects and revealed that a high proportion of participants exhibited patterns of both dorsal and anterior spreads. However, we observed that the unilateral ESPB was ineffective in alleviating the pain intensity after RFA of the hepatic tumour.

An increasing number of studies have investigated the spread patterns of ESPB in larger living cohorts [10,17,18]. For instance, Abdellav et al. assessed the injectate spread in 60 patients who received a T4 ESPB using CT imaging and reported a limited extent of both paravertebral and epidural spread [18]. The differences in ESPB injectate distribution within paravertebral space between Abdellav et al.’s study and this study may be attributed to variations in the injection site (T4 vs. T10) and the time interval between injection and CT imaging (15 min vs. 47 min). In contrast, Sørenstua et al. recently evaluated the spread pattern of ESPB in 10 healthy volunteers using 30 mL of diluted ropivacaine at the T7 level [10]. Their findings revealed paravertebral spread in 9 out of 10 participants, with a median spread of 4 levels; intercostal spread in all participants, with a median spread of 5.5 levels; and epidural spread in 4 out of 10 participants, with a median spread of 0 levels. These results align with the proportions of ESPB spread reported in this study. Additionally, Shan et al. assessed injectate spread in 84 patients who received a T7 ESPB under CT imaging and observed a similar extent of paravertebral and epidural spread as reported in our study [17]. By integrating the findings from Sørenstua et al. [10], Shan et al. [17] and our present study, we concluded that the drug spread patterns may be similar at the mid (T7) and low thoracic levels (T10) in living subjects. These findings of living subjects could be valuable in planning the execution of ESPB in clinical practices.

Compared to the aforementioned studies conducted in living subjects, this study differs in several aspects. Firstly, the CT-guided RFA model employed in this research minimized radiation exposure to the participants by utilizing low-dose CT scans. Secondly, in the three aforementioned studies conducted in living subjects, ESPB was performed in the prone position, whereas in this study, it was performed in the lateral position. Thirdly, additional analyses were conducted to examine the relationships between gender, muscle thickness, and injectate spread, which may provide insights into the differences between studies in living subjects and cadaveric studies. For instance, the anterior spreads of ESPB, including paravertebral, intercostal, and epidural spread, were not extensively reported in cadaveric studies. Among the 16 cadaveric studies on thoracic ESPB, only 11 found evidence of paravertebral dye penetration [3]. In addition, we observed wide cranio-caudal spread patterns, with a median spread across nine vertebral levels in this study. This spread pattern was found to be generally broader than those reported in cadaveric studies, which revealed a cranio-caudal spread of 3–6 vertebral levels [3]. Furthermore, we noted that the drug spread was more pronounced toward the cranial end compared to the caudal end, aligning with the direction of needle insertion. Live subjects’ fascial planes and compartments are significantly influenced by dynamic forces, as muscles and fasciae slide over each other, potentially contributing to a wider transmission of the ESPB drug injection’s pressure. Interestingly, we also observed a significantly negative correlation between the back muscle thickness and the extent of ESPB dorsal spread. This finding emphasizes the potential differences in drug spread patterns between cadaveric models and living subjects. It is worth noting that the observed median numbers of levels of intercostal spread and paravertebral spread in this study were lower than those reported in case series studies of living subjects who received ESPB injections at the same T10 level. For example, Schwartzmann et al. revealed a paravertebral spread of seven levels and an intercostal spread of six levels in a female patient after the T10 ESPB injection [19]. The same research group also reported injectate spreads of 9 [5–12] and 3 [2–6] levels to the intercostal space and neural foramina (similar to paravertebral spreads) in a series of six female patients after the T10 ESPB injection [20]. In contrast, our present study found that female patients may exhibit a broader intercostal spread following a T10 ESPB, while the patients in the above two studies were all female. It is essential to note that our study enrolled patients with hepatocellular cancer, and over 70% of them were male [21]. Since male subjects generally have higher muscle tone and back muscle thickness compared to female subjects [22,23], this factor might have an impact on the extent of ESPB spread patterns.

This study reported findings of analgesic effects that are discordant with two recent studies on ESPB administration for ablation of hepatic tumours. Mostafa et al. reported that ESPB alleviated post-procedural pain of radiofrequency ablation of hepatic tumours [9], while Gergin et al. also reported similar pain relief after microwave ablation of hepatic tumours [8]. These conflicting findings may be attributed to several reasons. Firstly, the procedures in our study included multiple tumour ablations, resulting in significantly longer average procedure times (approximately 70 min) compared to Mostafa et al.’s study (approximately 25 min) and Gergin et al.’s study (approximately 10 min). Secondly, while we applied a sham block in participants of this study, this was not performed in the other two studies. Thirdly, variations in hepatic tumour size may significantly influence both opioid requirements during RFA and post-procedural pain intensity. For instance, in the study by Gergin et al. hepatic tumours in the ESPB group were smaller than those in the control group, which may have contributed to differences in analgesic outcomes [8]. By contrast, patients in the ESPB group had statistically non-significantly larger tumour size than those in the sham group (approximately 1 cm larger) in this study. As a result, patients in the ESPB group in the study by Gergin et al. reported lower pain scores in the post-anaesthetic care unit, whereas patients in the ESPB group in this study required a higher, though not statistically significant, fentanyl dose during RFA. Fourthly, the patients in this study underwent complex RFA for hepatic tumours, with a more prolonged procedure time than Mostafa et al.’s and Gergin et al.’s studies [8,9], which could have resulted in a higher intensity of post-RFA visceral pain due to more hepatic tissue necrosis.

The unsatisfactory analgesic of unilateral ESPB to post-RFA pain may be because of several reasons. First, pain signals from the liver are primarily transmitted *via* visceral afferents, which travel alongside sympathetic nerves and enter the spinal dorsal horn at the T7–T12 levels. These nerve fibres predominantly pass through the prevertebral ganglia including celiac and splanchnic ganglion, and relay signals to the central nervous system *via* the splanchnic nerve [24]. Consequently, effective analgesia targeting these nerves requires deeper injections, such as a splanchnic nerve block [25,26] or epidural analgesia, to achieve adequate pain relief. Since the spread of local anaesthetics in ESPB primarily affects somatic nerves, as demonstrated in this study, its impact on visceral nerves is relatively limited. Consistently, we observed a low incidence of epidural spread, which may explain why ESPB failed to alleviate post-RFA visceral pain. Second, Sørenstua et al. recently reported discrepancies between what could have been expected from the images of ESPB injectate spread and the test result of cutaneous sensation blockade [10]. This study was compatible to the above literature regarding the discrepancy between injectate spread and the unsatisfactory analgesic effect. Several alternative techniques, beyond splanchnic nerve block and epidural analgesia, may improve post-RFA pain management. For instance, bilateral ESPB has been shown to provide superior analgesic effects, [27], and increasing the local anaesthetic concentration in ESPB may further enhance its efficacy [28].

This study has several limitations. Firstly, we did not conduct the sensory test due to the sham injection performed in this study. Performing the sensory test could have violated the blinding of randomization, given the use of a sham block. Moreover, previous reports indicate that sensory loss may not accurately reflect the actual spread of local anaesthetics [3,10], making it unreliable to confidently assume clinical effect based on sensory testing results [10]. Secondly, we observed that female patients had more intercostal spread than male patients. However, it is essential to note that this study was conducted during the SARS-CoV-2 pandemic, making patient enrolment challenging. Since the majority of patients with hepatocellular carcinoma were male [21], the sample size of our study included an insufficient number of female patients, and therefore, the study may have been underpowered to confirm any potential influence of gender on drug spread patterns. Thirdly, the CT image was performed approximately 47 min after the ESPB, whereas late diffusion of the injectate may occur around 60 min after ESPB injection, as previously reported [3].

## Conclusion

In conclusion, this study provided valuable insights into the drug spread patterns of right T10 ESPB, with a significant number of participants demonstrating both dorsal and anterior spreads. Additionally, we observed that the thickness of the back muscle and the sex of the participants may have an impact on the extent of ESPB spread. However, it is important to note that unilateral ESPB was found to be ineffective in alleviating the pain intensity after RFA of the hepatic tumour.

## Supplementary Material

Supplementary materials.doc

## Data Availability

The data that support the findings of this study are available from the corresponding author upon reasonable request. The data are not publicly available because of privacy and ethical restrictions.

## References

[1] Kot P, Rodriguez P, Granell M, et al. The erector spinae plane block: a narrative review. Korean J Anesthesiol. 2019;72(3):209–220. doi: 10.4097/kja.d.19.00012. 30886130 PMC6547235

[2] Kim SH. Anatomical classification and clinical application of thoracic paraspinal blocks. Korean J Anesthesiol. 2022;75(4):295–306. doi: 10.4097/kja.22138. 35368174 PMC9346276

[3] Chin KJ, El-Boghdadly K. Mechanisms of action of the erector spinae plane (ESP) block: a narrative review. Can J Anaesth. 2021;68(3):387–408. doi: 10.1007/s12630-020-01875-2. 33403545

[4] Giorgio A, Tarantino L, de Stefano G, et al. Complications after percutaneous saline-enhanced radiofrequency ablation of liver tumors: 3-year experience with 336 patients at a single center. AJR Am J Roentgenol. 2005;184(1):207–211. doi: 10.2214/ajr.184.1.01840207. 15615976

[5] Canıtez A, Kozanhan B, Aksoy N, et al. Effect of erector spinae plane block on the postoperative quality of recovery after laparoscopic cholecystectomy: a prospective double-blind study. Br J Anaesth. 2021;127(4):629–635. doi: 10.1016/j.bja.2021.06.030. 34340839

[6] Ibrahim M. Erector spinae plane block in laparoscopic cholecystectomy, is there a difference? a randomized controlled trial. Anesth Essays Res. 2020;14(1):119–126. doi: 10.4103/aer.AER_144_19. 32843804 PMC7428093

[7] Huang X, Wang J, Zhang J, et al. Ultrasound-guided erector spinae plane block improves analgesia after laparoscopic hepatectomy: a randomised controlled trial. Br J Anaesth. 2022;129(3):445–453. doi: 10.1016/j.bja.2022.05.013. 35803754

[8] Gergin ÖÖ, Pehlivan SS, Erkan İ, et al. Clinical efficacy of ultrasound guided erector spinae plane block in patients undergoing microwave ablation. Saudi Med J. 2022;43(9):1027–1034. doi: 10.15537/smj.2022.43.9.20220245. 36104059 PMC9987667

[9] Mostafa SF, El Mourad M. Ultrasound guided erector spinae plane block for percutaneous radiofrequency ablation of liver tumors. Egyptian Journal of Anaesthesia. 2020;36(1):305–311. doi: 10.1080/11101849.2020.1854156.

[10] Sørenstua M, Zantalis N, Raeder J, et al. Spread of local anesthetics after erector spinae plane block: an MRI study in healthy volunteers. Reg Anesth Pain Med. 2023;48(2):74–79. doi: 10.1136/rapm-2022-104012. 36351741

[11] Chin KJ, Lirk P, Hollmann MW, et al. Mechanisms of action of fascial plane blocks: a narrative review. Reg Anesth Pain Med. 2021;46(7):618–628. doi: 10.1136/rapm-2020-102305. 34145073

[12] Kallmes DF, Buchbinder R, Miller FG. Viewpoint: randomised controlled trials using invasive control interventions should be included in Cochrane Reviews. Cochrane Database Syst Rev. 2011;2011(8):ED000030. doi: 10.1002/14651858.ED000030. 21833988 PMC10846439

[13] Rahimzadeh P, Faiz SHR, Salehi S, et al. Unilateral right-sided ultrasound-guided erector spinae plane block for post-laparoscopic cholecystectomy analgesia: a randomized control trial. Anesth Pain Med. 2022;12(6):e132152. doi: 10.5812/aapm-132152. 36938107 PMC10016115

[14] Chung K, Choi ST, Jun EH, et al. Role of erector spinae plane block in controlling functional abdominal pain: case reports. Medicine (Baltimore). 2021;100(39):e27335. doi: 10.1097/MD.0000000000027335. 34596137 PMC8483877

[15] Lee B-C, Liu K-L, Wu C-H, et al. Comparison of radiofrequency ablation and transarterial chemoembolization for hepatocellular carcinoma in the caudate lobe. Cardiovasc Intervent Radiol. 2018;41(11):1699–1707. doi: 10.1007/s00270-018-1978-0. 29946941

[16] Myles PS, Shulman MA, Reilly J, et al. Measurement of quality of recovery after surgery using the 15-item quality of recovery scale: a systematic review and meta-analysis. Br J Anaesth. 2022;128(6):1029–1039. doi: 10.1016/j.bja.2022.03.009. 35430086

[17] Shan T, Zhang X, Zhao Z, et al. Spread of local anaesthetic after erector spinae plane block: a randomised, three-dimensional reconstruction, imaging study. Br J Anaesth. 2025;134(3):830–838. 39788818 10.1016/j.bja.2024.10.046 PMC11867096

[18] Abdella A, Arida E, Megahed NA, et al. Analgesia and spread of erector spinae plane block in breast cancer surgeries: a randomized controlled trial. BMC Anesthesiol. 2022;22(1):321. doi: 10.1186/s12871-022-01860-w. 36253729 PMC9575234

[19] Schwartzmann A, Peng P, Maciel MA, et al. Mechanism of the erector spinae plane block: insights from a magnetic resonance imaging study. Can J Anaesth. 2018;65(10):1165–1166. doi: 10.1007/s12630-018-1187-y. 30076575

[20] Schwartzmann A, Peng P, Maciel MA, et al. Magnetic resonance imaging study of local anesthetic spread in patients receiving an erector spinae plane block. Can J Anaesth. 2020;67(8):942–948. doi: 10.1007/s12630-020-01613-8. 32152885

[21] Wu EM, Wong LL, Hernandez BY, et al. Gender differences in hepatocellular cancer: disparities in nonalcoholic fatty liver disease/steatohepatitis and liver transplantation. Hepatoma Res. 2018;4:4. doi: 10.20517/2394-5079.2018.87. 30687780 PMC6347119

[22] Wu Z, Wang Y, Ye Z, et al. Effects of age and sex on properties of lumbar erector spinae in healthy people: preliminary results from a pilot study. Front Physiol. 2021;12:718068. doi: 10.3389/fphys.2021.718068. 34616306 PMC8488426

[23] Heizelmann A, Tasdemir S, Schmidberger J, et al. Measurements of the trapezius and erector spinae muscles using virtual touch imaging quantification ultrasound-Elastography: a cross section study. BMC Musculoskelet Disord. 2017;18(1):370. doi: 10.1186/s12891-017-1733-8. 28841869 PMC5574109

[24] Tan X, Sivakumar S, Bednarsch J, et al. Nerve fibers in the tumor microenvironment in neurotropic cancer-pancreatic cancer and cholangiocarcinoma. Oncogene. 2021;40(5):899–908. doi: 10.1038/s41388-020-01578-4. 33288884 PMC7862068

[25] Kapural L, Lee N, Badhey H, et al. Splanchnic block at T11 provides a longer relief than celiac plexus block from nonmalignant, chronic abdominal pain. Pain Manag. 2019;9(2):115–121. doi: 10.2217/pmt-2018-0056. 30681022

[26] Papadopoulos D, Kostopanagiotou G, Batistaki C. Bilateral thoracic splanchnic nerve radiofrequency thermocoagulation for the management of end-stage pancreatic abdominal cancer pain. Pain Physician. 2013;16(2):125–133. 23511679

[27] Cesur S, Y R Ko Lu HU, Aksu C, et al. Bilateral versus unilateral erector spinae plane block for postoperative analgesia in laparoscopic cholecystectomy: a randomized controlled study. Braz J Anesthesiol. 2023;73(1):72–77. doi: 10.1016/j.bjane.2021.04.020. 33932389 PMC9801199

[28] Altıparmak B, Korkmaz Toker M, Uysal Aİ, et al. Comparison of the efficacy of erector spinae plane block performed with different concentrations of bupivacaine on postoperative analgesia after mastectomy surgery: randomized, prospective, double blinded trial. BMC Anesthesiol. 2019;19(1):31. doi: 10.1186/s12871-019-0700-3. 30832580 PMC6399855

